# Design of a Portable Microfluidic Platform for EGOT-Based in Liquid Biosensing

**DOI:** 10.3390/s22030969

**Published:** 2022-01-26

**Authors:** Matteo Segantini, Matteo Parmeggiani, Alberto Ballesio, Gianluca Palmara, Francesca Frascella, Simone Luigi Marasso, Matteo Cocuzza

**Affiliations:** 1 Department of Applied Science and Technology (DISAT), Politecnico di Torino, Corso Duca degli Abruzzi 24, 10129 Torino, Italy; matteo.segantini@polito.it (M.S.); alberto.ballesio@polito.it (A.B.); gianluca.palmara@polito.it (G.P.); francesca.frascella@polito.it (F.F.); simone.marasso@polito.it (S.L.M.); matteo.cocuzza@infm.polito.it (M.C.); 2 CNR-IMEM, Parco Area delle Scienze, 37a, 43124 Parma, Italy

**Keywords:** microfluidics, EGOFETs, OECTs, simulations, biosensors

## Abstract

In biosensing applications, the exploitation of organic transistors gated via a liquid electrolyte has increased in the last years thanks to their enormous advantages in terms of sensitivity, low cost and power consumption. However, a practical aspect limiting the use of these devices in real applications is the contamination of the organic material, which represents an obstacle for the realization of a portable sensing platform based on electrolyte-gated organic transistors (EGOTs). In this work, a novel contamination-free microfluidic platform allowing differential measurements is presented and validated through finite element modeling simulations. The proposed design allows the exposure of the sensing electrode without contaminating the EGOT device during the whole sensing tests protocol. Furthermore, the platform is exploited to perform the detection of bovine serum albumin (BSA) as a validation test for the introduced differential protocol, demonstrating the capability to detect BSA at 1 pM concentration. The lack of contamination and the differential measurements provided in this work can be the first steps towards the realization of a reliable EGOT-based portable sensing instrument.

## 1. Introduction

In recent years several microfluidic platforms have been proposed for applications ranging from lab-on-a-chip (LOC) biosensors [1], to characterization of liquids [2], genetic applications [3], and energy production in fuel cells [4,5]. The main advantages of microfluidics-based LOC biosensors reside in their inherent portability for on-field measurements, and low sample consumption. Among the different kinds of biosensors, electrolyte-gated organic transistors (EGOTs) are emerging as promising devices due to their low limit of detection, biocompatibility and low fabrication costs [6]. Different approaches have been proposed in the literature for the integration of such devices in microfluidic platforms. Asano et al. demonstrated the real time detection of glyphosate in water exploiting a microfluidics integrated EGOT [7], while White et al. developed a versatile floating gate transistor for the label-free detection of DNA [8] and proteins [9].

EGOTs are three terminal devices in which the current between two electrodes (i.e., source and drain) can be modulated through the application of a voltage via the third electrode (i.e., gate). As their name suggests, these devices are made of an organic material that is usually a semiconductive polymer. The most interesting feature of EGOTs is that, differently from conventional organic field-effect transistors (OFETs), they are coupled with the gate through an electrolyte that can be either solid or liquid. When using organic semiconductors (OSCs) in direct contact with an electrolyte two different operation modes are possible. If the OSC is permeable to ions and capable to withstand mixed electronic/ionic conduction, an electrochemical operation is obtained [10,11]. In this case the electrochemical doping/dedoping of the semiconductor controls the charge carrier density in the device channel, and the device is an organic electrochemical transistor (OECT). When instead the OSC is not permeable to ions, an electrical double layer (EDL) is formed at the semiconductor/electrolyte interface as well as between the electrolyte and the polarizable gate electrode (or only at the OSC/electrolyte interface if a non-polarizable electrode is exploited). In this case, the voltage drop at the solid/liquid interface allows controlling the charge carrier density, and an electrolyte gated organic field effect transistor (EGOFET) is obtained [12]. These EDLs are associated with high capacitances, up to three orders of magnitude greater than the capacitances associated to conventional OFET [13]. An even higher effective surface capacitance is associated to the operation of OECTs, where the bulk of the film contributes to the accumulation of charges [14]. Thus, EGOTs are extremely sensitive to any change occurring at one of the two above-mentioned surfaces, which can be exploited to use these devices as sensors [15,16,17,18,19,20,21,22]. In fact, it is possible to modify the surface of the gate electrode with a biofunctionalization in order to bind specifically any target of interest. Once a specific binding event occurs, the properties of the gate electrode surface change with a corresponding shift of the output current of the transistor. In this way, it is possible to associate the shift of the output signal with the amount of analyte present in the electrolytic solution, allowing for a quantification of the tested biomolecule. EGOTs have already been proven to allow detection of low concentrations of different biomolecules (i.e., proteins [20,23,24], drugs [25,26], or ribonucleic acids [27,28]), achieving limits of detection that can permit the use of these device in real applications (i.e., lab-on-chip) [18].

However, in all these studies there is a practical impediment that could be an obstacle towards the realization of a portable sensing platform, which is the contamination of the organic material. As a matter of fact, during sensing tests involving EGOT-based devices the binding of the analyte to the gate surface is usually made manually in an external reservoir, which makes impossible to translate this protocol into an integrated and automated platform (i.e., lab-on-chip) [21]. The reason behind the external incubation of the biomolecules deals with the possible contamination of the OSC material. In fact, being the analyte free to move in the electrolytic solution, it is possible that part of the analyte is adsorbed in a non-specific way onto the sensor surface, changing in this way the output of the transistor. Washing protocols can be applied to mitigate this problem, but still the organic polymer can suffer of unwanted and non-reversible doping effects due to the exposure to the analyte [13,29,30]. A promising approach to avoid OSC contaminations has been proposed by White et al. [8,9], which exploited an extended-gate architecture in order to separate the sensing region (i.e., the biofunctionalized gate electrode) from the electronic transducer (i.e., the EGOT). This approach however requires additional fabrication steps to deposit and pattern the ion-gel in order to couple the organic transistor with the sensing electrode, and the increased number of EDL capacitances in series may be expected to reduce the overall sensitivity of the device when compared to non-extended gate EGOFETs, which demonstrated limit of detection down to a single molecule [21]. In this work a new approach is proposed to integrate the biorecognition step into a sensing platform, with a particular focus on the simulation-assisted design process. In addition to this, a method based on differential measurements is presented, which allows more reliable results in EGOT biosensing tests. A microfluidic chip is designed with three separate chambers, one hosting the sensor with the OSC material, one containing the functionalized gate electrode and another one for the reference electrode. Two inlets are present, one dedicated to the solution containing the analyte and one for the blank electrolytic buffer. Thanks to this design, it is possible to flow the analyte only towards the functionalized gate chamber, without contaminations of the EGOT. In order to validate the proposed design, finite element modeling simulations are performed exploiting Comsol Multiphysics*^®^* software. The simulations account for both the convection and diffusion of the biomolecules of interest and they demonstrate that the proposed design impedes the analyte to enter the biosensor chamber during the injection phase, as well as it avoids the biomolecule diffusion onto the EGOT during the incubation phase. The simulations also show the efficiency of the washing step, which completely removes all the unbound molecules from the functionalized gate chamber.

Finally, the detection of bovine serum albumin (BSA) by differential measurements is reported as a further validation of the integrated microfluidic platform. BSA is a protein commonly exploited as a passivating agent to prevent non-specific binding during biosensing experiments. Being well characterized in the literature and inexpensive, it was chosen here as a target to provide an economical proof of concept for the presented platform. The results also represent a proof of principle for the new differential method introduced for EGOT biosensing, which minimizes the effect of the sensor signal drift with a dramatic improvement of the device sensing capabilities.

## 2. Materials and Methods

### 2.1. Finite Element Simulations

The simulations were performed using COMSOL Multiphysics^®^ (COMSOL AB, Stockholm, Sweden) The geometry used in the simulation was directly imported from the same computer-aided design (created with Rhinoceros^®^, Robert McNeel & Associates, Seattle, WA, USA) used to fabricate the final prototype exploited in the sensing test. A 2D model was used in this work to decrease the computational cost of the simulations and the study was run in the time-dependent mode, with a free triangular-based mesh of the geometry. The reference pressure and temperature for this study were set respectively to 1 atm and 293.15 K.

Since the model involved the motion of particles in a liquid, two software built-in physics have been exploited: the Laminar Flow and Transport of Diluted Species. The Laminar Flow physics was used to study single-phase fluid flows with low Reynold’s number (i.e., lower than 2000). This physics accounts for the solution of the Navier-Stokes equation for the conservation of momentum and the continuity equation for the conservation of mass for incompressible fluids, as reported in Equations (1) and (2):

(1) $$\rho \frac{\partial u}{\partial t}+\rho \left(u\cdot \nabla \right)u=-\nabla p+\mu \nabla^{2}u+F$$

(2) $$\rho \nabla \cdot \left(u\right)=0$$

where *u* is the field velocity, *ρ* is the fluid density, *μ* is the fluid dynamic viscosity and *F* represents the external forces applied to the fluid. In order to mimic the same conditions present during the sensing test, step functions have been used to describe the flow velocity profile of both the analyte and the washing buffer (see Table 1). The velocity value of 1.7 × 10^−3^ m/s was calculated considering a flow rate of 200 µL/min and a channel section of 1 mm^2^.

The Transport of Diluted Species physics has been chosen to compute the motion of the biomolecules diluted in the main buffer. It was imposed that the diluted species can move only through diffusion (according to Flick’s law) and convection (coupled with the Laminar Flow physics). The overall equation to be solved is reported in Equation (3):

(3) $$\frac{\partial c}{\partial t}-\nabla \cdot J+u\cdot \nabla c=R$$

where *c* is the concentration of the diluted species, *J* is the total flux and *R* is the volumetric source of the concentration. An incoming flux from the analyte inlet is set to a fixed concentration of 150 × 10^−9^ mol/m^3^, which corresponds to a concentration of 150 pM in 1 mL of solution (the same used in the sensing test).

### 2.2. Sensing Platform Fabrication

#### 2.2.1. Microfluidics

The microfluidics was realized in polydimethylsiloxane (PDMS) through replica molding starting from two masters, one defining the microfluidic channels and the opening windows and another one used as sealing cap. The opening windows define the exposed area of both the sensor and the gates and they were set to 9 mm^2^ for both of them. The two masters were fabricated with Vero White photosensitive resin (Stratasys, Ltd., Eden Prairie, MN, USA) by means of a 3D printer (Objet30 by Stratasys^®^, Stratasys, Ltd., Eden Prairie, MN, USA). After a thorough cleaning in water to remove the support material, they were kept in oven at 110 °C for 12 h, which is a fundamental step to allow the following PDMS polymerization [31]. A final wash in acetone (5 min in ultrasonic bath) completed the fabrication process of the two masters. The PDMS (Sylgard™ 184, Dow Chemical Company, Midland, MI, USA, 10 parts of elastomer for one part of curing agent) was then casted into the masters and kept in oven for 2 h for the polymerization.

To avoid any chemical bonding of the PDMS and to ensure a tight sealing of the microfluidics, two holders in polymethyl methacrylate (PMMA) were fabricated. They were patterned with a *CO_2_* laser cutting machine (Microla Optoelectronics Srl, Chivasso, Italy) to define the housings for the gate electrodes and the biosensor, as well as the holes for the screws used to seal and auto-align the whole platform.

#### 2.2.2. EGOT

Cleanroom processes were exploited for the fabrication of the biosensor. In particular, two lift-off processes were used to pattern both the gold electrodes and the aluminum oxide passivation layer.

Firstly, the Ti Prime adhesion promoter (MicroChemicals GmbH, Ulm, Germany) was spin-coated (4000 rpm, 30 s) onto a p-doped silicon wafer terminated with a 1 µm thick *SiO_2_* layer and baked at 120 °C for 2 min. Then, the *AZ 5214 E* image reversal photoresist was spin-coated (4000 rpm, 30 s), followed by a soft bake (105 °C, 2 min). The photoresist was then UV-exposed for 6.5 s (8 mW/cm^2^) with the specific mask for the transfer of the desired pattern. A reversal bake was performed (105 °C, 2 min), after which the photoresist was again UV-exposed (21.5 s, 8 mW/cm^2^, flood exposure). The unwanted photoresist was finally removed with the AZ 726 MIF developer (MicroChemicals GmbH, Ulm, Germany). At this point, a 10 nm thick layer of *Ti* followed by 100 nm thick layer of *Au* were e-beam evaporated onto the wafer. The first lift-off process was then finalized by rinsing the wafer in acetone for 30 min. The obtained transistor channel length and width were 9960 µm and 10 µm, respectively (Figure S1, Supplementary Materials). The second lift-off process was used to pattern the passivation layer, which is fundamental to avoid parasitic currents. The process was carried out following the same protocol described above except for the e-beam evaporation, which was performed by depositing 150 nm of *Al_2_O_3_*. The wafer was then diced (Microla Optoelectronics Srl) and 36 chips were obtained from one single wafer. The dimensions of each chip were 12.8 mm (width) and 11.8 mm (length).

The polymer used for the biosensing test was poly [3-(5-carboxypentyl)thiophene-2,5-diyl] (P3CPT), whose behavior in EGOT is known to be dominated by the electrochemical operation mode [32,33]. The deposition process of the polymer followed a well-established protocol exploited in other works of our group [34]. P3CPT was dissolved in dimethyl sulfoxide (DMSO) at 2.5 mg/mL concentration and left stirring at 50 °C for 1 h to allow for a complete dissolution. Once cooled down, the solution containing the polymer was spin-coated onto the chip (50 µL, 2000 rpm for 30 s) and then annealed in dynamic vacuum for 2 h at 75 °C.

### 2.3. Biosensing Test

#### 2.3.1. Measurement Setup

The setup used for the characterization of the biosensor was composed by a probe station, a source measure unit (SMU) and two external pumps. The probe station ensured the electrical contact with the biosensor via three micromanipulators (one for each transistor terminal) connected to the B2912A SMU (Keysight Technologies, Santa Rosa, CA, USA). A common source configuration was used: the first channel of the SMU was dedicated to the drain-to-source connection, while the second channel was exploited for the gate-to-source coupling. A peristaltic pump (IPC4 from ISMATEC^®^, Wertheim, USA) was used to deliver the analyte, while a motorized syringe (NE-4000 Double Syringe Pump from KF Technology, Roma, Italy) was exploited for the delivery of the main buffer, which was phosphate buffered saline (PBS) 1×.

#### 2.3.2. EGOT Characterization

To quantify the presence of the biomolecule (bovine serum albumin, BSA), transfer characteristics curves of the EGOT were recorded. The transfer characteristic (*I_D_-V_G_*) consists in monitoring the channel current (*I_D_*) while varying the gate voltage (*V_G_*) with a fixed drain voltage (*V_D_*). The *V_G_* was swept between 0.1 V and −0.6 V and the *V_D_* was kept at −0.4 V. The sweeping on the gate voltage was made through a pulsed protocol that reduces the amount of electrical bias stress applied to the polymer [35]. The scan rate used for this measurement was 40 mV/s.

From the *I_D_-V_G_* curves it is possible to extract two figures of merit (FoM) of the transistor, which are exploited for the assessment of the analyte concentration. The two FoM are the threshold voltage *V_T_* and the maximum transconductance *G_max_* and they can be calculated starting from the current *I_D_*. In the saturation regime, the *I_D_* current can be written as (Equation (4)):

(4) $$\left|I_{D}\right|=\frac{W}{2L}\mu C_{T}{\left(\left|V_{G}\left|-\right|V_{T}\right|\right)}^{2}\;$$

where *W* and *L* are respectively the transistor channel width and length, *µ* is the carriers mobility and *C_T_* is the total capacitance, which results from the series of the capacitances associated to the EDL present at the gate/electrolyte interface and the channel capacitance of the device [36]. The two FoM can be then calculated as:

(5) $$\left|V_{T}\right|=|V_{G}|-\sqrt{\frac{2L\left|I_{DS}\right|}{W\mu C_{T}}}\;$$

(6) $$G_{max}=\max\left(\frac{\partial I_{D}}{\partial V_{G}}\right)=\max\left(\frac{W}{L}\mu C_{T}\left(|V_{G}\left|-|V_{T}\right|\right)\right)$$

According to Equation (5) the threshold voltage of the devices is obtained as the intercept on the abscissa axis of the linear fit of the square root of *I_DS_*. The quantification of the analyte is made through a differential measurement, which means that each FoM is measured both with the reference as well as the functionalized electrode. Being *F* the FoM of interest, its value at a specific analyte concentration (*F_c_*) is calculated as:

(7) $$F_{c}=|F_{c}^{Fun}\left|-\right|F_{c}^{Ref}|$$

where *F_c_^Fun^* and *F_c_^Ref^* are the FoM calculated at the analyte concentration *c* for the functionalized and reference gate, respectively. In this way, the variation of the FoM at the different analyte concentrations can be calculated as:

(8) $$\Delta F_{c}=\frac{F_{c}-F_{0}}{\left|F_{0}\right|}$$

where *F*_0_ is the differential FoM at the beginning of the experiment (i.e., without analyte).

#### 2.3.3. Gate Functionalization

The gate electrodes were obtained by e-beam evaporation of 10 nm of *Ti* and 100 nm of Au on a SiO_2_-terminated silicon wafer. The wafer was then diced (Microla Optoelectronics Srl) in order to obtain a rectangular gate electrode with dimensions 7.8 mm × 11.8 mm.

The electrodes were washed in ethanol, followed by a quick rinse in 2-propanol and dried under a stream of nitrogen. A thiol solution consisting of 10 mM 3-mercaptopropionic acid (3-MPA) dissolved in ethanol was prepared and used for incubating the samples for 1 h at room temperature on an orbital shaker. After the formation of the self-assembled monolayer (SAM) of thiols on Au surfaces, the samples were washed twice in fresh ethanol and once in ultrapure water (UPW). Finally, they were dried under a nitrogen stream.

The exposed terminal carboxyl groups on the electrodes surface were activated by means of a carbodiimide crosslinking chemistry, in order to make them more reactive towards primary amines, as described in a previous work [37]. Briefly, the samples were incubated 15 min in a solution of 50 mM 2-(N-morpholino)ethanesulfonic acid (MES buffer) followed by another 15 min incubation in a solution of 4/10 mM 1-ethyl-3-(3-dimethylaminopropyl)carbodiimide (EDC)/N-hydroxysulfosuccinimide (Sulfo-NHS) in MES buffer. Lastly, the samples were rinsed 3 times for 5 min in Phosphate Buffered Saline (PBS) on an orbital shaker and dried under a stream of nitrogen.

Each electrode was incubated with 80 µL of a solution of 1 µg/mL anti-BSA antibody in PBS at 4 °C overnight. After that, three washing steps of 5 min in Tween^®^ 20 supplemented PBS (PBS-tween) 0.05% (*v*/*v*) on an orbital shaker were performed. Finally, the electrodes were dried under a nitrogen stream and used for the measurements.

#### 2.3.4. Sensing Protocol

Firstly, the main buffer was injected throughout the microfluidic platform with the motorized syringe with a flow rate of 200 µL/min. After that, an *I_D_-V_G_* curve was recorded both on the functionalized and reference gate to extract the starting value (i.e., without analyte) of the main FoM of the transistor.

The analyte (dissolved in PBS 1×) is then injected in the microfluidic system with the peristaltic pump with a flow rate of 200 µL/min for 10 min. The flow is subsequently stopped for 15 min to allow the incubation of the analyte onto the functionalized gate surface. Finally, the blank buffer is again flowed in with the motorized syringe at 200 µL/min for 15 min to wash away all the unbound excess of the analyte. At this point, another *I_D_-V_G_* curve is recorded and the new values of the FoM are registered. In order to produce a calibration curve, growing concentrations of the analyte are tested applying the same procedure described above. The studied concentrations of BSA were 1 pM, 15 pM, 30 pM and 150 pM.

## 3. Results

### 3.1. Microfluidic Design

**Figure 1:**
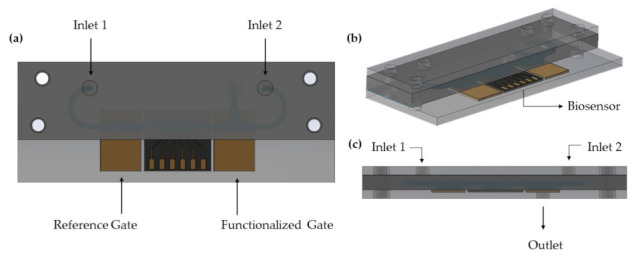
Top (**a**), perspective (**b**) and side (**c**) view of the microfluidic platform hosting two gold electrodes (reference and functionalized gates) and the EGOT biosensor.

The proposed design of the microfluidic platform is presented in Figure 1.

The microfluidic platform is composed of two gold electrodes, the EGOT biosensor, the PDMS microfluidic channels and two PMMA holders, which are held together by the use of four screws.

In this configuration two inlets are present, one dedicated to the blank buffer (*Inlet 1*) and one for the analyte (*Inlet 2*). Three different chambers are present in this design, one for the reference electrode, one for the biosensor and another one for the functionalized electrode. This last chamber also hosts the outlet channel.

The core concept behind this design is the allocation of the lowest fluidic resistance of the system in correspondence of the functionalized gate chamber. The latter is in fact designed in a T-shape structure in which two sides have a much larger fluidic resistance than the third one, being the outlet channel. Considering the center of the functionalized gate chamber, from left (*Inlet 1*) and right (*Inlet 2*) the high fluidic resistances arise from the channel restrictions that have been expressly designed, so that any flow coming from *Inlet 1* or *Inlet 2* is more likely to flow towards the outlet channel. It should be also considered that the microfluidic platform is linked to the external pumps through microtubes. Thus, the hydrostatic pressure plays a fundamental role in increasing the fluidic resistance values coming from the two inlets.

This considered, when the analyte is injected from *Inlet 2* it will pass through the functionalized gate chamber and eventually flow out from the outlet channel. In this way the gate can be exposed with the right amount of analyte, while the biosensor is not contaminated because there is no backflow towards *Inlet 1*. The same happens during the wash phase, in which the blank buffer passes throughout the gate chamber towards the outlet.

### 3.2. Finite Element Simulation

The simulation with the finite element modeling of the proposed design was needed in order to validate the effective absence of contamination of the biosensor. In fact, despite the microfluidic platform was designed according to the principle of the fluid dynamics, it was also necessary to take into account the aspects related to the diffusion in liquid of the biomolecules. This is particularly important for the incubation phase, during which all the fluids are at rest for 15 min and only diffusion phenomena driven by the concentration gradient occur. In addition, the simulation can provide a solid confirmation of the effectiveness of the design by validating the fluidic part as well.

To model the diffusion of the biomolecule, it was necessary to provide the model with the diffusion coefficient of the analyte of interest (i.e., BSA). To this aim, the protein diffusion can be easily calculated by exploiting the Stokes-Einstein equation, which holds for spherical particles in liquid at low Reynold’s number:

(9) $$D=\frac{k_{B}T}{6\pi \eta r}$$

where *D* is the diffusion coefficient, *k_B_* is the Boltzmann constant, *T* is the absolute temperature, *η* is the dynamic viscosity of the fluid and *r* is the radius of the particle. This equation is valid by assuming the protein as a spherical particle, which is true in a first approximation for folded proteins [38].

In this case, the molecular weight of the BSA is 66 kDa, which is associated to a hydrodynamic radius of 3.48 nm in the folded status [39,40]. Considering the PBS dynamic viscosity of 1 mPa·s and an absolute temperature of 293.15 K, from Equation (9) it is possible to find the value of the diffusion coefficient of BSA in PBS buffer, which is equal to 6.2 × 10^−11^ m^2^/s [41]. This value is consistent with other data available in literature, which demonstrates how this approach can be easily extended even for those biomolecules for which there are not documented data about their diffusivity [42,43].

**Figure 2:**
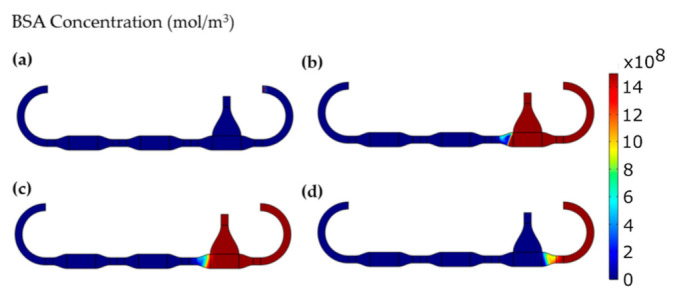
BSA concentration during the different steps of the sensing protocol: (**a**) at the beginning, (**b**) after analyte injection, (**c**) after incubation, (**d**) after wash.

Once the calculated data were fed into the software, an entire sensing protocol was simulated. The obtained concentration of BSA at the different steps of the protocol is reported in Figure 2 and in Video S1 (Supplementary Materials).

At the beginning (Figure 2a) there are not BSA molecules inside the microfluidic chamber, which is all filled with the blank buffer. When the injection of the analyte is completed (Figure 2b) the BSA effectively flowed just through the functionalized gate electrode chamber, preventing any back flow towards the biosensor. This result represents the first proof of the validity of the design, which does not allow the EGOT contamination when the analyte moves by convection inside the microfluidics.

**Figure 3:**
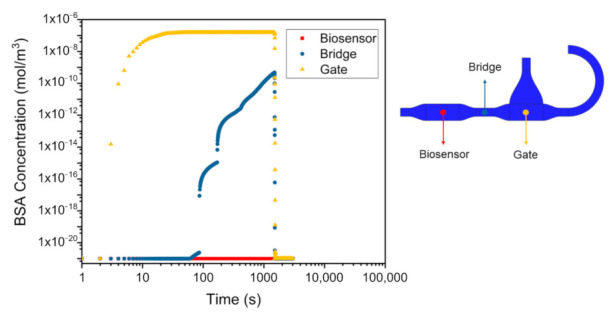
BSA concentration evolution in three different points of the geometry: the functionalized gate electrode chamber, the bridge and the biosensor chamber.

After 15 min of incubation (Figure 2c) the simulation shows the occurrence of a diffusion process that displaced some of the BSA molecules towards the biosensor. However, the simulation also proves that the diffusion dynamics of BSA molecules diluted in PBS is slow enough to prevent the transistor contamination to occur when exploiting this microfluidic geometry. Further details of the BSA diffusion are reported in Figure 3, in which the time evolution of the analyte concentration is shown for three different points, such as the functionalized gate chamber, the biosensor chamber and the center of the channel dividing those two chambers (from here on called *bridge*). For the sake of clarity, the graph was obtained by setting the concentration of the blank buffer as 1 × 10^−21^ mol/m^3^, which is to be considered the reference value when there is not BSA in the system.

This result quantitatively shows the validity of the proposed microfluidic platform, given that the analyte concentration in the biosensor chamber is always at the reference value.

Considering the bridge, one can observe that the concentration of the BSA rapidly increases during the first minutes, after which it follows the typical diffusion dynamics (i.e., square root behavior in time). Interestingly, diffusion starts dominating the BSA concentration variation already when the analyte is still flowing. This result justifies the use of simulation software for microfluidic design purpose, since it demonstrated that diffusion phenomena can be fundamental for the assessment of the actual concentration of the analyte, even when dealing with molecules with relatively small diffusion coefficients (as it is for BSA).

Finally, the simulation demonstrates the efficacy of the washing step as reported in Figure 2d and Figure 3. Not only the blank buffer completely removes any excess of the unbound BSA, but also the diffusion of the analyte from *Inlet 2* is avoided in the subsequent 10 min, which is the time needed to record the sensor output.

### 3.3. Biosensing Test

**Figure 4:**
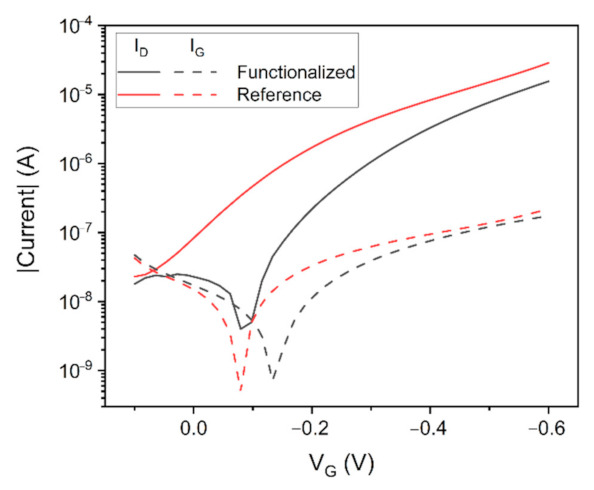
Transfer characteristic curves recorded with *V_D_* = −0.4 V in PBS 1×. Solid lines represent the channel current *I_D_* while dashed lines represent the gate current *I_G_*.

The sensing test was carried out by injecting growing concentrations of BSA according to the experimental protocol described in the Materials and Methods section. Firstly, DC output curves were recorded to evaluate the biosensors conductivity (Figure S2, Supplementary Material), which was assessed to 4.4 ± 0.4 mS/V. After that, the EGOT was characterized with the blank buffer and the performance of the two gate electrodes (i.e., with and without functionalization) was recorded. The transfer characteristic curves of the transistor are reported in Figure 4.

The first thing to notice is the greater absolute value of the channel current *I_D_* when recorded through the reference gate electrode. According to Equation (4) this difference can be ascribed either to a change of the threshold voltage value or the system capacitance (or to a combination of the two), since the mobility does not change being the polymer always the same. Considering the measured hole mobility of 5.23 × 10^−4^ cm^2^/Vs (Figure S3, Supplementary Materials), from Equations (5) and (6) it is possible to extract the EGOT characteristics for both the gate electrodes. As reported in Table 2, the transistor has a lower threshold voltage in module and a greater maximum transconductance with the reference electrode, which is mainly due to the higher effective surface capacitance *C_T_*. Considering the measured polymer film thickness of 40 nm, from *C_T_* it is possible to extract a volumetric capacitance of 130 F/cm^3^ and 220 F/cm^3^ with the functionalized and reference electrode respectively, in agreement with values found in the literature for other similar polymers exploited in OECTs [14]. The values of the gate currents do not show great differences between the two electrodes.

**Figure 5:**
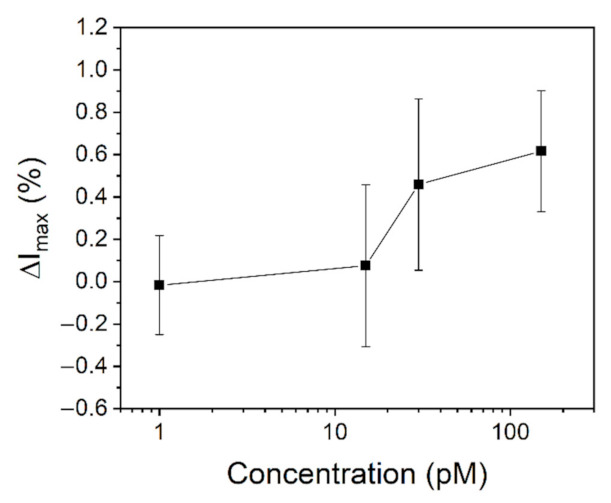
Differential channel current variation with the BSA concentration in PBS 1×. The error bars are calculated on at least three different devices.

After this initial characterization, the output signal difference is then monitored for the different concentrations of BSA. The obtained differential current *I_D_* variation is reported in Figure 5. The results show that the differential channel current increases with the BSA concentration, with a maximum variation registered at around 60% with respect to the starting level for a concentration of 150 pM of analyte. The sensitivity of the EGOT is maximized in the range between 15 pM and 50 pM of concentrations, in which the greatest variation of the output signal is recorded.

**Figure 6:**
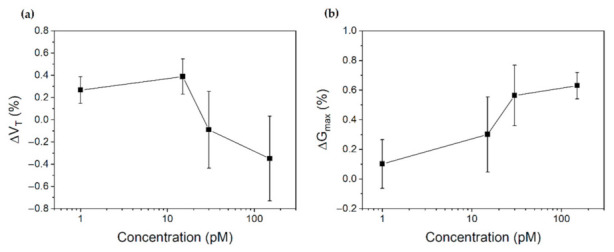
Differential variation of (**a**) the threshold voltage and (**b**) the maximum transconductance. The error bars are calculated on at least three different devices.

To have a clearer view of the effects of the BSA onto the sensor performance, the results were analysed in terms of *V_T_* and *G_max_* differential variations and reported in Figure 6.

The definition of the method to calculate the differential FoM presented in the Materials and Methods section makes the interpretation of these graphs and their correlation with the functionalized gate electrode extremely easy. As a matter of fact a positive variation in the graph means that the absolute value of the FoM under consideration is increasing. According to these definitions, it is then possible to notice that the threshold voltage has not a unique behavior with the concentration: *V_T_* in fact increases in module for 1 pM and 15 pM of BSA, while it rapidly decreases for concentrations greater than 50 pM. Conversely, *G_max_* is always increasing through the whole set of tested concentrations.

## 4. Discussion

### 4.1. Design and Simulation

The finite element simulations showed how the proposed design can be exploited to run biosensing protocols with the in-situ recognition of the analyte. From Figure 3 it is in fact evident that the BSA never reached the chamber hosting the EGOT biosensor.

Beyond the validation of the microfluidic platform, the obtained results also suggest the use of this simulation-based design as a template to build analyte-specific microfluidics. As a matter of fact, the contamination of the biosensor may happen through diffusion phenomena in those cases in which there is a biomolecule with great diffusivity, as well as when exploiting buffers with relatively small dynamic viscosities. As the reported results suggest, the majority of the diffusion effects are visible in the bridge section, being the microfluidic channel separating the biosensor and the functionalized gate. Thus, it is possible to exploit this simulation as a tool in which the bridge length is a parameter that changes as a function of the system diffusion coefficient. Knowing the biomolecule and fluid properties, thanks to this model it is then possible to calculate the minimum bridge length that ensure the absence of contaminations. This choice eventually optimizes the system in terms of total exploited volume of the fluid, which is fundamental when dealing with low concentrations in real samples.

Another important aspect to be considered is that this design can be potentially exploited also to run the in-situ functionalization of the gate electrode [44]. This realization would be a fundamental step towards the commercial exploitation of this sensing technologies, since it would overcome the practical limitation of a pre-functionalization of the gate electrode, with the consequent stability and stocking issues [45,46].

### 4.2. BSA Detection

One of the main achievements presented in this work is the methodology for the analyte quantification using differential measurements. In fact, it is known that EGOTs suffer from poor stability over time, which is one of the causes that limits their use in real applications [47,48]. The exploitation of the differential measurements can instead tackle this issue since each drift of the signal given by the OSC instability is reflected on both the electrodes and so eliminated.

Another important aspect to be taken into account is related to which FoM should be considered for the quantification of the analyte. According to the results presented in Figure 5 and Figure 6, it is in fact evident that the analysis of the output current is a less sensitive method compared to the analysis of the maximum transconductance. More specifically, by exploiting *I_D_* variations the minimum detected concentration was 15 pM, while the analysis of the differential *G_max_* revealed a variation of the signal already at 1 pM of concentration. The reason behind this difference can be understood according to the different mechanisms governing the detection of an analyte in EGOT biosensors. It is in fact known that the presence of a biolayer on the gate electrode surface can introduce either an additional polarization of the gate (in case the biomolecule is partially charged) or the change of the surface capacitance (or a combination of the two). The induced polarization is usually associated to a change in the threshold voltage, given that the additional charges of the biomolecule contribute to the accumulation (or reduction) of the available carriers in the OSC. On the contrary, the change of capacitance is usually associated to a change of the transistor transconductance. This interpretation of the effects of the analyte binding is complicated by the fact that *V_T_* and *G_max_* are not independent variables, so that any variation of one FoM partially affects the other one. In light of these considerations, it is possible to conclude that the FoM to be considered depends on which mechanism dominates the sensing in the system under analysis.

From the study of the FoM presented in Figure 6, it is possible to state that the main physical mechanism dominating the sensing of BSA in PBS 1x is related to the change of the system capacitance. In particular, the binding of the BSA molecules causes an increase of the overall capacitance *C_T_*, which is reflected in the increase of the differential maximum transconductance. Interestingly, the capacitive effects are also dominating the threshold voltage for low BSA concentrations (i.e., up to 15 pM), since *V_T_* is increasing with *C_T_* accordingly to Equation (5). This result explains the limited sensitivity of the total current: the initial increase of *V_T_* in module compensates the increase of *G_max_* for a net change of the output current equal to zero. For concentrations greater than 15 pM, the increase of *G_max_* is instead high enough to control the total current growth, which is further enhanced by the decrease of the threshold voltage absolute value associated to an increase of negative charges (BSA isoelectric point is assessed around 4.7, thus it is negatively charged at PBS pH of 7.3 [49]).

## 5. Conclusions

In this work the design of a contamination-free microfluidic platform was proposed for the realization of a proof of concept of a portable sensing unit based on EGOTs. The lack of contamination of the biosensor was reached by designing a low fluidic resistance channel in correspondence of the functionalized gate chamber.

Finite element simulations were performed to prove the working principle of the designed microfluidics and to investigate the effects of the biomolecules diffusion. Considering bovine serum albumin (BSA) in phosphate buffered saline (PBS) 1×, the simulations proved that the biomolecules did not reach the biosensor during an entire sensing protocol. In addition, the simulations were proven to be a useful tool to design analyte-specific microfluidics that can take into account the system diffusion coefficient to calibrate the geometrical dimensions.

Finally, a prototype of the platform was realized and used to specifically detect BSA. The introduction of the differential measurements allowed the detection of the analyte at 1 pM concentration. The analysis of the figures of merit of the transistor revealed that the sensing mechanism for this case was mainly capacitive, allowing the recognition of the analyte even in a high ionic strength buffer.

## Figures and Tables

**Table 1:** Scheme of the velocity profile in time for the inlet with the analyte and the inlet containing the washing buffer.

Time (min)		Flow Velocity Target (m/s)	Flow Velocity Wash (m/s)	Description
0	10	1.7 × 10^−3^	0	Analyte injection
10	25	0	0	Incubation
25	40	0	1.7 × 10^−3^	Washing

**Table 2:** EGOT electrical characteristics measured with a bare (Reference) and Functionalized gold gate electrode.

Electrode	*|I_D_^max^|*(µA)	*V_T_* (mV)	*G_max_* (µS)	*C_T_* (µF/cm^2^) *
Functionalized	15	−244	97	523
Reference	26	−193	187	882

* Effective surface capacitance.

## Data Availability

The data presented in this study are available on request from the corresponding author.

## Supplementary Materials

The following supporting information can be downloaded at: https://www.mdpi.com/article/10.3390/s22030969/s1, Figure S1: EGOT Channel Dimensions; Figure S2: DC output curves; Figure S3: Time of Flight Measurements 10; Video S1: Simulated Sensing Protocol.
