# Supplementary material for: Design of a Portable Microfluidic Platform for EGOT-Based in Liquid Biosensing
Source: Sensors (Basel). 2022 Jan 26;22(3):969. doi: 10.3390/s22030969 (PMC8839715; doi:10.3390/s22030969)
Supplement: Supplementary file 1 [file sensors-22-00969-s001.zip › supplementary.pdf]

sensors

MDPI

Supplementary Materials

EGOT Channel Dimensions

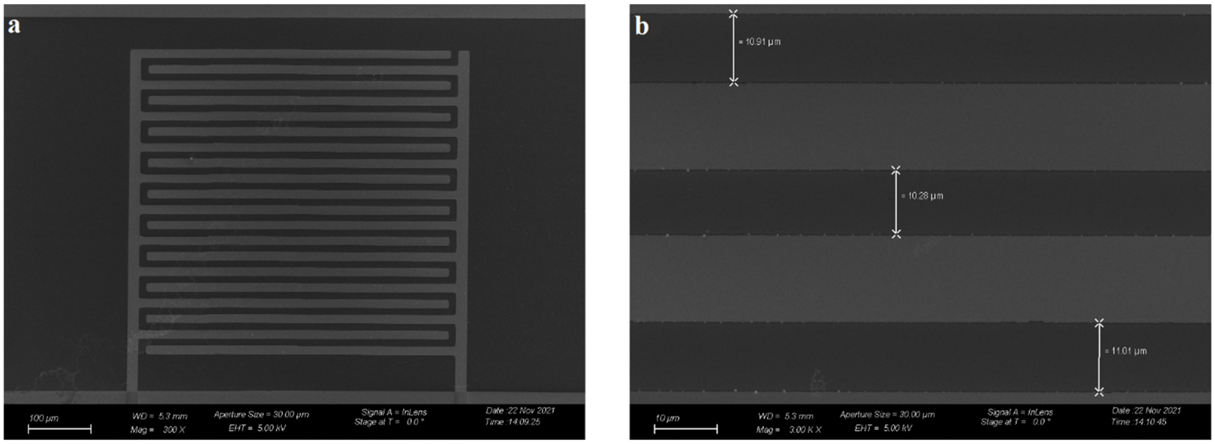

Figure S1. FESEM images of the interdigitated electrodes exploited for the realization of the EGOT-based biosensor. The average measured channel length is 10.7 nm.

DC Output Curves

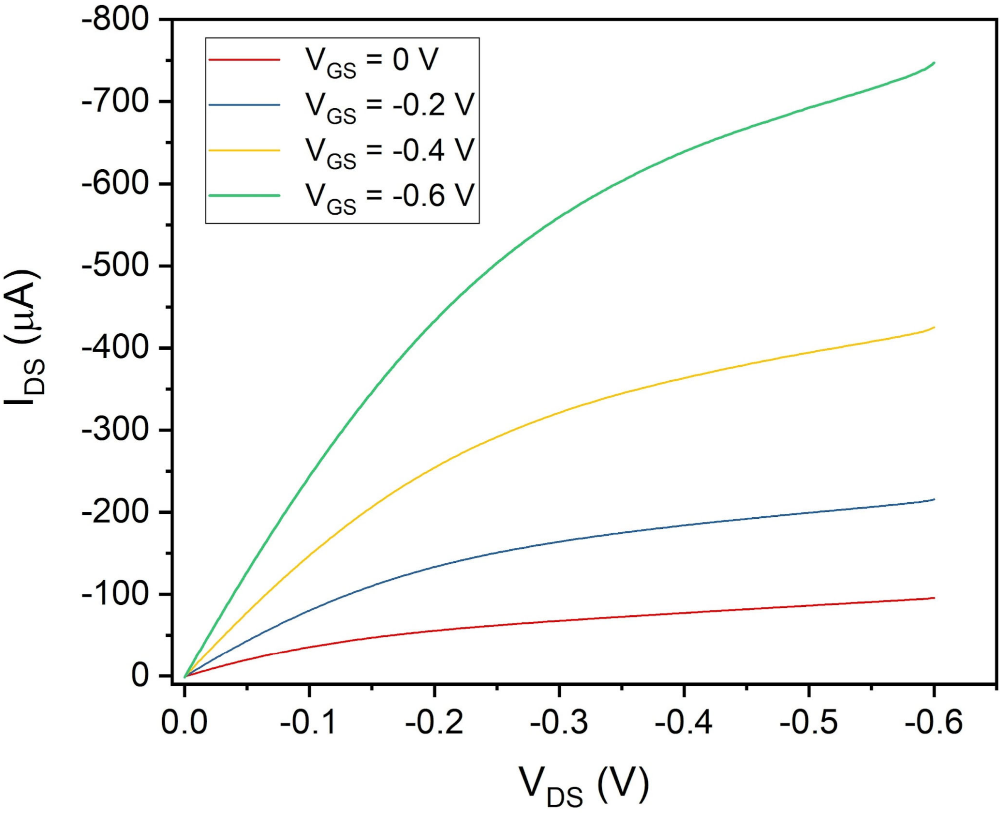

Figure S2. DC output characteristic curves recorded at different gate voltages with the reference electrode.

The conductivity of the fabricated sensors was investigated through the output curves. The drain to source voltage was varied from 0 V to –0.6 V (with 40 mV/s scan rate) for different values of the gate to source voltage (0 V, –0.2 V, –0.4 V and –0.6V). The gate voltage-normalized maximum conductivity was 4.4 ± 0.4 mS/V.

Time of Flight Measurements

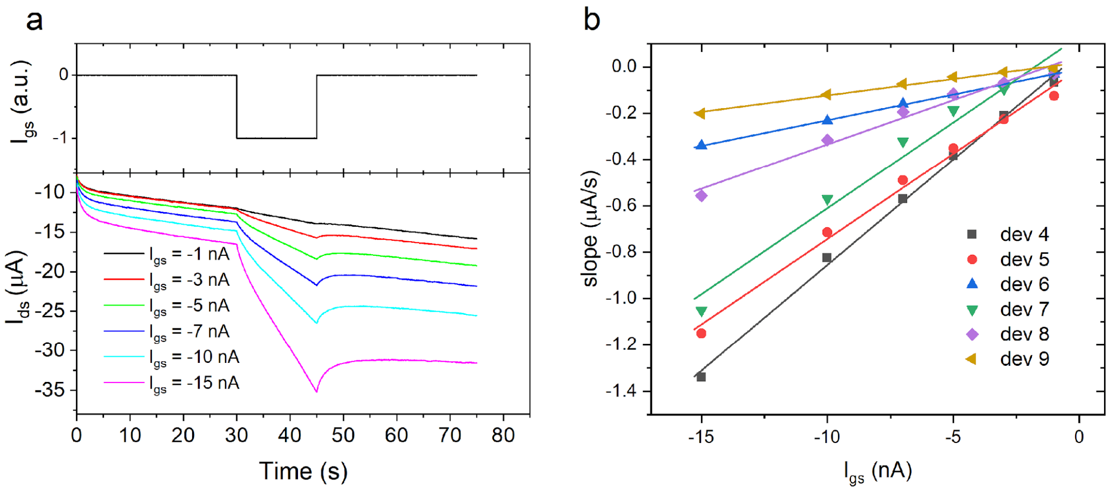

Figure S3. a) Time of flight measurement: device driven by a constant gate current with application of a 15 s pulse, all measurements performed at fixed V_{ds} = –100 mV. b) Linear fit of the slope $\frac{\partial I_{ds}}{\partial t}$ vs I_{gs} for six different devices.

The hole mobility for P3CPT based OECTs has been characterized by means of Time Of Flight measurements [1]. Figure S3 a show the response of I_{ds} to different I_{gs} pulses. By fitting the slope $\frac{\partial I_{ds}}{\partial t}$ vs I_{gs} (Figure S3, b) it is possible to extract the time of flight of holes across the transistor channel. The mobility is then obtained through the equation:

$$\mu_h = \frac{L^2}{\tau_h \cdot |V_{ds}|} \qquad \text{(S1)}$$

The average mobility obtained from 6 P3CPTs based devices was μ_h = (5.23 ± 3.17)·10^{-4} cm^2/Vs.

Reference

1. Bernards, D.A.; Malliaras, G.G. Steady-state and transient behavior of organic electrochemical transistors. *Adv. Funct. Mater.* **2007**, *17*, 3538–3544, doi:10.1002/adfm.200601239.
